# One-stage or two-stage revision surgery for prosthetic hip joint infection – the INFORM trial: a study protocol for a randomised controlled trial

**DOI:** 10.1186/s13063-016-1213-8

**Published:** 2016-02-17

**Authors:** Simon Strange, Michael R. Whitehouse, Andrew D. Beswick, Tim Board, Amanda Burston, Ben Burston, Fran E. Carroll, Paul Dieppe, Kirsty Garfield, Rachael Gooberman-Hill, Stephen Jones, Setor Kunutsor, Athene Lane, Erik Lenguerrand, Alasdair MacGowan, Andrew Moore, Sian Noble, Joanne Simon, Ian Stockley, Adrian H. Taylor, Andrew Toms, Jason Webb, John-Paul Whittaker, Matthew Wilson, Vikki Wylde, Ashley W. Blom

**Affiliations:** Musculoskeletal Research Unit, School of Clinical Sciences, University of Bristol, Level 1 Learning and Research, Southmead Hospital, Bristol, BS10 5NB UK; Wrightington Hospital, Wigan and Leigh NHS Foundation Trust, Hall Lane, Appley Bridge, Wrightington, Lancashire, WN6 9EP UK; The Robert Jones and Agnes Hunt Orthopaedic Hospital NHS Foundation, Oswestry, Shropshire SY10 7AG UK; School of Social and Community Medicine, University of Bristol, Canynge Hall, 39 Whatley Road, Bristol, BS8 2PS UK; University of Exeter, St Luke’s Campus, Heavitree Road, Exeter, EX1 2LU UK; Cardiff and Vale University Health Board, University Hospital Llandough, Penlan Road, Llandough, CF64 2XX UK; Bristol Randomised Trials Collaboration, University of Bristol, Canynge Hall, 39 Whatley Road, Bristol, BS8 2PS UK; North Bristol NHS Trust, Brunel Building, Southmead Hospital, Westbury-on-Trym, Bristol, BS10 5NB ᅟ; Sheffield Teaching Hospitals NHS Foundation Trust, Northern General Hospital, Herries Road, Sheffield, S5 7AU UK; Oxford University Hospitals NHS Foundation Trust, Nuffield Orthopaedic Centre, Windmill Road, Headington, Oxford, OX3 7HE UK; Royal Devon and Exeter NHS Foundation Trust, Barrack Rd, Exeter, Devon, EX2 5DW UK

**Keywords:** Infection, Hip replacement, Revision, One-stage, Two-stage, Patient-reported outcome measures, Randomised controlled trial, Cost-effectiveness

## Abstract

**Background:**

Periprosthetic joint infection (PJI) affects approximately 1 % of patients following total hip replacement (THR) and often results in severe physical and emotional suffering. Current surgical treatment options are debridement, antibiotics and implant retention; revision THR; excision of the joint and amputation. Revision surgery can be done as either a one-stage or two-stage operation. Both types of surgery are well-established practice in the NHS and result in similar rates of re-infection, but little is known about the impact of these treatments from the patient’s perspective. The main aim of this randomised controlled trial is to determine whether there is a difference in patient-reported outcome measures 18 months after randomisation for one-stage or two-stage revision surgery.

**Methods/Design:**

INFORM (INFection ORthopaedic Management) is an open, two-arm, multi-centre, randomised, superiority trial. We aim to randomise 148 patients with eligible PJI of the hip from approximately seven secondary care NHS orthopaedic units from across England and Wales. Patients will be randomised via a web-based system to receive either a one-stage revision or a two-stage revision THR. Blinding is not possible due to the nature of the intervention. All patients will be followed up for 18 months.

The primary outcome is the WOMAC Index, which assesses hip pain, function and stiffness, collected by questionnaire at 18 months. Secondary outcomes include the following: cost-effectiveness, complications, re-infection rates, objective hip function assessment and quality of life. A nested qualitative study will explore patients’ and surgeons’ experiences, including their views about trial participation and randomisation.

**Discussion:**

INFORM is the first ever randomised trial to compare two widely accepted surgical interventions for the treatment of PJI: one-stage and two-stage revision THR. The results of the trial will benefit patients in the future as the main focus is on patient-reported outcomes: pain, function and wellbeing in the long term. Patients state that these outcomes are more important than those that are clinically derived (such as re-infection) and have been commonly used in previous non-randomised studies.

Results from the INFORM trial will also benefit clinicians and NHS managers by enabling the comparison of these key interventions in terms of patients’ complication rates, health and social resource use and their overall cost-effectiveness.

**Trial registration:**

Current controlled trials ISRCTN10956306  (registered on 29 January 2015); UKCRN ID 18159.

**Electronic supplementary material:**

The online version of this article (doi:10.1186/s13063-016-1213-8) contains supplementary material, which is available to authorized users.

## Background

Total hip replacement (THR) is a highly successful treatment for painful and damaged joints, with over 80,000 primary THRs carried out in England, Wales and Northern Ireland in 2013 [1]. Periprosthetic joint infection (PJI) is an uncommon but serious complication, affecting approximately 1 % of patients who undergo primary THR and is the indication for over 1,000 revision procedures each year in the National Health Services of England and Wales (NHS) [1, 2].

PJI occurring within 2 years of THR is mainly surgically acquired, and is associated with joint pain and restricted movement. Early infections are commonly caused by virulent bacteria and cause acute onset of pain, effusion, erythema and fever. Delayed infections typically present with symptoms similar to aseptic joint failure including implant loosening and joint pain. If untreated, PJI can result in severe pain, restricted movement, disability and death [3].

Treatment options for hip PJI are the following: surgical removal of devitalised, damaged and infected tissue (debridement) with prosthesis retention and long-term antibiotic treatment; one-stage revision; two-stage revision; excision or amputation. Surgical debridement and retention is considered in early PJI with pathogens susceptible to antibiotics and in patients unfit for revision THR. However, this approach may require lifelong antibiotic treatment [4].

Surgical revision for a hip PJI involves prosthesis removal, debridement, antibiotic treatment and revision THR. The prosthesis is replaced in the same operation (one-stage) or replaced at a delayed interval of between 2 weeks and 12 months (two-stage). In a two-stage revision a temporary ‘spacer’ or temporary joint replacement may be fitted, but the patient has no definitive THR until it is replaced in the second operation. In England, Wales and Northern Ireland in 2013, treatments were one-stage (36 %), two-stage (60 %) and excision (4 %) [1].

The best treatment option is unclear. Two-stage revision has the potential for additional antimicrobial treatment and strategies, but patients’ mobility and quality of life are poor between stages [5, 6]. One-stage revision is becoming increasingly popular because compared with two-stage revision, it has the potential to reduce the overall burden on patients of major surgery, and to reduce healthcare costs [7].

To compare outcomes of one-stage and two-stage revision of infected hip replacements, we systematically reviewed studies that included populations representative of patients in routine clinical practice. Irrespective of the surgical treatment used, the overall 2-year rate of re-infection was 10.1 % (95 % CI 8.2–12.0). In 11 studies with 1225 patients with a hip PJI receiving exclusively one-stage revision, the rate of re-infection at 2 years was 8.6 % (95 % CI 4.5–13.9). After two-stage revision exclusively in 28 studies with 1188 patients, the rate of re-infection at 2 years was 10.2 % (95 % CI 7.7–12.9). We conclude that on the basis of a systematic review of published data, there is no difference in the re-infection rate between one-stage and two-stage revision THR for PJI [8].

### Rationale for the trial

Currently, both one-stage and two-stage revision THRs are carried out for hip PJI. Surgeons from the collaborating centres are in agreement that a proportion of patients could be treated with either one-stage or two-stage revision and that there is no definitive evidence to recommend a specific strategy in terms of clinical or patient outcomes [9].

Unlike previous studies on infection after joint replacement, we plan to use patient-centred outcome measures rather than re-infection rates. This is because patient and public involvement conducted at the lead study centre during the development of this trial protocol, as well as previous research around outcomes after surgery, show that patients are concerned about pain, function and wellbeing after surgery, rather than a single bio-medically defined outcome [10, 11]. This is particularly relevant to PJI as treatment appears to be distressing and has a substantial impact on quality of life [5, 9]. Qualitative research will form an integral part of this trial, informing the design and development of trial processes such as recruitment and randomisation, and to facilitate the interpretation of the trial findings.

#### Null hypothesis

There is no difference in patient-reported outcomes (as measured by the Western Ontario and McMaster Universities Arthritis (WOMAC) Index) at 18 months post randomisation, after one-stage or two-stage revision THR, for PJI.

## Methods/Design

The Infection Orthopaedic Management (INFORM) study is a pragmatic, multi-centre two-armed, parallel group, open, randomised, superiority trial with 1:1 allocation and a nested qualitative study.

The study obtained ethical approval from NRES Committee South West – Frenchay on 31 December 2014 (14/SW/1166).

### Study setting

Patients will be initially recruited from seven NHS secondary care orthopaedic units in England and Wales. If necessary, the trial will be enlarged to more centres to achieve the recruitment target. Selected sites are high-volume tertiary referral centres for infected joint replacements or large NHS orthopaedic units. Participating surgeons at each centre have experience and expertise in both one-stage and two-stage revision treatment.

### Study duration

Recruitment into the trial commenced in March 2015 and 18-month follow-up for all participants is anticipated to be completed by August 2018.

### Participants

Patients will be eligible for the study if they have a hip PJI, and are deemed suitable for either one-stage or two-stage revision surgery by their treating surgeon. The diagnosis of infection, monitoring and decision to proceed to revision surgery, will be determined by the treating surgeon, or multidisciplinary team at the unit. This pragmatic approach should mean that the results of the trial are generalisable to the wider population of patients with this condition.

Patients will provide their written, informed consent to participation before entering the study.

#### Inclusion criteria

- Aged 18 years or above
- A clinical diagnosis of hip PJI
  - o Diagnosis will be guided by clinical, haematological, biochemical, microbiological and radiological findings from investigations prior to surgery, in accordance with local procedures and at the discretion of the treating clinical team
- Require revision surgery (either one-stage or two-stage revision THR) for hip PJI in the opinion of the treating consultant orthopaedic surgeon(s)

#### Exclusion criteria

- Unable or unwilling to undergo either one-stage or two-stage revision surgery
- Lacking capacity to give written informed consent for research

### Interventions

#### One-stage revision THR

One operation: the infected prosthetic joint is removed along with any potentially infected materials, the surgical site is debrided, irrigated and a new THR is implanted under the same anaesthetic.

#### Two-stage revision THR

Two operations: in the first operation, the infected prosthetic joint is removed along with any potentially infected materials and the surgical site is debrided and irrigated. In a second operation, under a separate anaesthetic, a new THR is implanted. The antibiotic regime between stages (e.g. duration, route) will be prescribed according to local guidelines at each centre. The delivery of local antibiotics and the use of a static or articulating spacer will be determined by the treating surgeon depending upon intra-operative findings at the time of the first surgical intervention.

At the time of surgical intervention(s), all cases will have tissue samples collected from five different sites with clean instruments to allow an adequate number and quality of samples to be available for microbiological testing [12].

All other aspects of treatment (e.g. clinical investigations; surgical approach; pre-, peri- and post-operative antibiotic regimens; choice of implants and fixation; analgesia) will be according to the treating surgeon’s normal practice and in line with local policies and procedures.

#### Follow-up

Patients will remain in routine clinical follow-up, with the frequency, duration and clinicians present (e.g. consultant surgeon, microbiologist), determined by local resources and clinical need. Response to treatment will be monitored by blood tests (e.g. C-reactive protein) as clinically indicated and the occurrence of re-infection will be determined by the presenting clinical history and signs as elicited by the treating clinical team, consistent with the preoperative diagnosis of infection.

Research assessments will take place preoperatively (prior to one-stage or first of a two-stage revision), and then every 3 months until 18 months post-randomisation. Outcomes will be assessed using self-report questionnaires, a clinical performance test and extraction of data from medical records (Additional file 1: Figure S1).

#### Safety

Both interventions in the INFORM trial are common surgical procedures for treating hip PJI in the NHS. Surgeons from the centres participating in the trial are experienced in these procedures and specialise in treating PJI. There are no additional risks to patients in taking part in the trial as the clinical interventions and follow-up can be considered standard care. Regular monitoring of outcomes may be perceived as a benefit to some. Patients will be informed of the risks of the operation during the surgical consent process, as is standard practice.

All adverse events will be recorded and serious adverse events will be notified to the appropriate authorities (Research Ethics Committee and Sponsor) within specified timelines.

#### Outcome measures

### Primary outcome measure

The WOMAC Index measured at 18 months post-randomisation.

The WOMAC Index is a validated patient-reported outcome measure, widely used in THR research [13]. The index consists of 24 items (5 pain, 2 stiffness and 17 physical function) divided into three subscales and can be completed in less than 12 minutes. Response options are in a 5-point Likert scale format and the index is validated for completion on site or over the telephone [14].

#### Secondary outcome measures

Complications relating to the study:Complications relating to the study such as hip dislocation, deep vein thrombosis/pulmonary embolism, nerve damage, additional surgery, hospital readmissions, length of stay and re-infection will be collected from hospital medical records and by telephone call or personal visit to participants, every 3 months post-randomisation for 18 months.

#### Post-operative pain

The Brief Pain Inventory short form (BPI-sf) is a validated, widely used, self-administered questionnaire which measures both the intensity of pain (sensory dimension) and interference of pain in the patient’s life (reactive dimension). Patients will complete the eleven questions (four on pain severity, seven on pain interference) rated on a scale of 0–10. Other non-compulsory items have been omitted [15].

#### Hip function

The Oxford Hip Score (OHS) is a short, self-administered questionnaire, which has been validated for use in THR. It consists of 12 questions about activities of daily living directly affected by poor hip function [16].

The 20-metre timed walk test will be used as an objective measure of hip function. This will be performed in hospital preoperatively and at 18 months post randomisation [17].

#### Quality of life and mental wellbeing

The EuroQol EQ-5D-5 L is a validated quality of life measure, consisting a descriptive system (five dimensions; each dimension having five levels) and a visual analogue scale (patient’s self-rated health recorded on a 20-cm scale) [18].

The Hip Dysfunction and Osteoarthritis Outcome Score (HOOS), quality of life subscale consists of four questions each on a 5-point Likert scale with each question being scored from 0 to 4. A normalised score (100 indicating no symptoms and 0 indicating extreme symptoms) is calculated from the subscale. This instrument is specifically designed to capture how the patient’s hip symptoms impact on their lifestyle [19].

The Hospital Anxiety and Depression Scale (HADS) is a validated self-reported measure used to detect anxiety and depression in people with physical health problems. The HADS comprises two subscales, depression and anxiety. Each subscale has a score ranging from 0 to 21. Items are rated on a 4-point Likert-type scale ranging from 0 to 3, generating a scale range of 0 to 42 points, with higher scores representing greater symptom severity [20].

#### Cost-effectiveness

All health service resource use relating to the hip PJI will be collected from hospital records and from patient self-completed questionnaires. This will include the interventions, additional inpatient stays, outpatient appointments and any related surgical or non-surgical procedures. Questionnaires will collect information on non-treating hospital resource use, community health and social service use, travel costs, time off work and informal care. Resource use logs, to act as an aide memoire, will be given to patients at their initial preoperative assessment to help them complete the follow-up questionnaires.

### Sample size

The required sample size has been set at 128 participants; allowing for a 13 % loss to follow-up at 18 months post randomisation, a total of 148 patients will need to be recruited. To reach this target, we will need to identify 290 eligible patients: the recruitment rate observed in a surgical trial involving THR recently conducted in the coordinating centre was 51 % with an attrition rate of 13 % [21].

A sample size of 128 patients will provide 80 % power to test that one surgical approach is superior to the other approach 18 months post randomisation by 10 points on the WOMAC Index, equivalent to a 0.5 standard deviation difference. The significance level for this superiority hypothesis is set at 5 % (two-sided).

Although it is known that infection following total joint replacement reduces patient satisfaction and seriously impairs functional health and quality of life, there is no published research on the likely difference in patient-reported outcomes between patients undergoing one-stage and two-stage revision for PJI. The standard deviations observed prior to one-stage or two-stage revision surgery for WOMAC global and sub-indices range between 18 and 25 [22, 23].

### Randomisation

After patients have been consented to the trial and have agreed to be randomised, preoperative outcome measures will be collected. They will then be randomly allocated to one of the two treatment groups (one-stage or two-stage revision surgery) in a 1:1 ratio. Randomisation within blocks of varying size will be conducted separately for each hospital. Block sizes will not be disclosed.

Randomisation will take place as close as possible to the time of surgery (maximum 12 weeks prior to surgery), whilst allowing sufficient time to plan the operation and order necessary equipment.

The randomisation sequence will be generated centrally by computer, and administered via the Internet by the Bristol Randomised Trial Collaboration.

### Blinding

Due to the nature of the interventions, it is not possible to blind patients or staff to allocation. Patients will be informed of the allocated surgery after they have completed the preoperative assessments.

### Statistical analysis

Analysis and presentation of data will be in accordance with the Consolidated Standards of Reporting Trials (CONSORT) guidelines for reporting randomised trials, and the final report will also follow the CONSORT extension for non-pharmacological interventions (see Additional file 1: Figure S1 – study flowchart). The baseline characteristics and primary outcomes will be described by treatment group. The secondary outcomes will also be presented by treatment group and where required by assessment point. Means with standard deviation or median with inter-quartile range will be reported for continuous variable, frequency and proportions for categorical or binary variables, as appropriate.

The primary outcome is the continuous WOMAC Index collected at 18 months postrandomisation. The primary analysis will consist of a generalised linear mixed model (GLLM) with identity link function regressing baseline and 18-month WOMAC measures on the treatment indicator (one-stage treatment versus two-stage treatment), measurement point (baseline versus 18-months) and their interaction (treatment × measurement point). This will be a two-level hierarchical GLLM (measurements within patients). As the treatment allocation is stratified by hospitals and participants are nested within a small number of hospitals, the primary analysis will also be adjusted for indicators of hospital centre introduced as fixed effects (using the coordination centre as a reference). The intervention effect will be assessed using appropriate contrast to identify the mean difference in WOMAC at 18 months and due emphasis will be put on the associated 95 % confidence interval and the *p* value resulting from the Wald-test associated with this contrast. The primary analysis will be based on the intention-to-treat principle, analysing participants in the groups to which they were randomised. All individuals with a WOMAC Index observed at baseline and/or at 18 months will be considered for this analysis. The GLLM with identity link function is based on a likelihood method, which provides unbiased estimation under the missing at random hypothesis despite the presence of missing outcome information. Various sensitivity analyses will be conducted to adjust for imbalance baseline characteristics between treatment groups, assess the clustering at surgeon level or investigate the impact of missing data using various imputation strategies. Finally a per-protocol analysis will be conducted.

Secondary analyses will firstly analyse all repeated measurements of WOMAC to compare the trajectory of recovery/change between treatment groups. Similarly to the analyses of the WOMAC Index, we will firstly investigate the differences in OHS, HADS and 20-metre timed walk test between treatment groups at 18 months post randomisation (using a time × treatment group interaction and appropriate contrast). We will then analyse all repeated measurements of those secondary outcomes to compare the trajectory of recovery/change between treatment groups. GLLMs with appropriate link function will be used (using, according to the nature of the outcomes, linear, logistic or Poisson two- or three-levels mixed regression) to assess the difference between treatments in secondary outcomes and take into account data clustering. Length of hospital stay will be assessed and compared using generalised linear (Poisson or negative-binomial) or survival analysis model. The trial is not primarily powered for these analyses and the results will be interpreted with due caution.

### Health economic analysis

We will conduct an intention-to-treat cost-effectiveness analysis from a societal perspective with costs to the NHS reported and analysed separately. All costs will be reported in 2018/2019 prices, and discounting will be applied as appropriate.

Health service resource use will be valued using hospital finance department and routine UK data [24, 25]. Social service, patient and informal carer resource use will be valued using routine [25, 26] and self-reported data.

The net monetary benefit statistic, using the difference in costs and the difference in quality-adjusted life years (QALY) between groups, and adjusted for hospital centre, baseline values (e.g. preoperative WOMAC, EQ-5D-5 L) and any covariate imbalance, will be calculated for different values of societal willingness to pay for a QALY. This will be the primary economic analysis.

The secondary economic analysis will examine the difference in costs with the differences in the WOMAC Index. If no arm is dominant, i.e. does not have statistically significant improved WOMAC Index and lower costs, then an incremental cost-effectiveness ratio will be estimated and cost-effectiveness acceptability curves will be derived using bootstrapping techniques. These will show the probability of the intervention being cost-effective at a range of ‘willingness to pay’ thresholds.

Sensitivity analysis will account for uncertainty and imprecision in measurements including multiple imputation models for missing values.

### Nested qualitative study

During the trial we will conduct a qualitative interview study to explore patients’ experiences of taking part in the trial, their treatment for PJI and their recovery. The interviews will focus on the acceptability of the interventions and patients’ experiences during the follow-up period. Issues around mobility and return to function, complications, expectations and perceptions of how they feel that their treatment could be improved (if at all) as well as any challenges faced during the recovery period will be discussed.

We will interview up to 40 patients at two time points: post intervention and at the end of the study (approximately 18 months later). We will also interview up to 20 surgeons participating in the trial to explore the acceptability of the trial recruitment and randomisation processes.

Findings from these interviews will help to refine trial processes and inform an understanding of people’s experiences and perceptions of the interventions and the care they received.

The interviews will be audio-recorded, fully transcribed, anonymised and analysed cross-sectionally and longitudinally using framework method and constant comparison.

## Discussion

This paper describes a multi-centre randomised trial to compare two widely accepted surgical interventions to treat infected prosthetic hip joint replacements. The aim is to establish the most patient-focussed and cost-effective surgery to treat this devastating condition.

Randomised controlled trials (RCTs) are the highest level of evidence available to assess the effectiveness of surgical interventions. Despite this, there is a paucity of robust, appropriately powered trials generally, and in the field of orthopaedic surgical interventions specifically, that utilise appropriate methodology. Whilst the nature of the intervention (i.e. a different number of operations being performed) means it is not possible to effectively blind participants or surgeons to the intervention being used, we have been able to address other common problems observed in surgical RCTs in our design. These include the lack of an a *priori* sample size calculation, poorly defined inclusion and exclusion criteria, the use of unvalidated outcome measures and lack of detail of the statistical analysis employed [27–30]. The INFORM trial will present generalisable data to support patient, clinician and healthcare decision-making in the treatment of patients with PJI of the hip.

### Trial status

The INFORM trial received permission to conduct research at the lead centre on 19 January 2015. The first participant was enrolled on 4 March 2015.

## Additional file

Additional file 1: Figure S1. Consort flow diagram. A visual representation of the pathway of patients through the trial. (DOC 39 kb)

## Abbreviations

BPI-sf: Brief Pain Inventory short-form
HADS: Hospital Anxiety and Depression Scale
HOOS: Hip Dysfunction and Osteoarthritis Outcome Score
OHS: Oxford Hip Score
PJI: prosthetic joint infection
QALY: quality-adjusted life year
RCT: randomised controlled trial
THR: total hip replacement
WOMAC: Western Ontario and McMaster Universities Arthritis Index
