# Supplementary material for: One-stage or two-stage revision surgery for prosthetic hip joint infection – the INFORM trial: a study protocol for a randomised controlled trial
Source: Trials. 2016 Feb 17;17:90. doi: 10.1186/s13063-016-1213-8 (PMC4756538; doi:10.1186/s13063-016-1213-8)
Supplement: Additional file 1: Figure S1. — Consort flow diagram. A visual representation of the pathway of patients through the trial. (DOC 39 kb) [file 13063_2016_1213_MOESM1_ESM.doc]

**Allocation**

**Enrollment**

Eligible: Patients with Prosthetic Hip Joint Infection deemed clinically suitable for either One-stage or Two-stage revision surgery, by their treating surgeon

Primary, Secondary and Cost-effectiveness analyses.

Description of serious adverse events and complications by treatment group

Excluded:

♦  Under 18 years

♦  Decline to participate

♦  Lack capacity to give written

informed consent for research

Randomised (n=148)

One-stage revision (n=74)

Two-stage revision (n=74)

Pre-Operative and regular assessments up to 18 months:

- WOMAC (patient reported outcomes)
- EQ-5D-5L (health economics)
- BPI (self reported pain)
- OHS (self reported hip function)
- HADS (self reported anxiety and depression)
- HOOS (self reported quality of life)
- 20-metre timed walk (performance test)
- Complications, including reinfection
- Health service resource use
- Interviews: Post-op and 18 months

**Follow-Up**

Pre-Operative and regular assessments up to 18 months:

- WOMAC (patient reported outcomes)
- EQ-5D-5L (health economics)
- BPI (self reported pain)
- OHS (self reported hip function)
- HADS (self reported anxiety and depression)
- HOOS (self reported quality of life)
- 20-metre timed walk (performance test)
- Complications, including reinfection
- Healthcare service resource use
- Interviews: Post-op and 18 months

**Analysis**

Figure 1
